# Comparison of the clinical and sonographic effects of ultrasound therapy, extracorporeal shock wave therapy, and Kinesio taping in lateral epicondylitis

**DOI:** 10.3906/sag-2001-79

**Published:** 2021-02-26

**Authors:** Tarık ÖZMEN, Salih Süha KOPARAL, Özlem KARATAŞ, Filiz ESER, Bülent ÖZKURT, Ümit GAFUROĞLU

**Affiliations:** 1 Department of Physiotherapy and Rehabilitation, Faculty of Health Sciences, Karabük University, Karabük Turkey; 2 Department of Radiology, Ankara Numune Training and Research Hospital, Ankara Turkey; 3 Department of Physical Medicine and Rehabilitation, University of Health Sciences, Antalya Training and Research Hospital, Antalya Turkey; 4 Department of Physical Medicine and Rehabilitation, Ankara Numune Training and Research Hospital, Ankara Turkey; 5 Department of Orthopedics, Ankara Numune Training and Research Hospital, Ankara Turkey

**Keywords:** Extracorporeal shock wave treatment, lateral epicondylitis, taping, ultrasonography

## Abstract

**Background/aim:**

The aim of this study was to compare the clinical and sonographic effects of the ultrasound (US) therapy, extracorporeal shock wave therapy (ESWT), and Kinesio taping (KT) in the lateral epicondylitis (LE).

**Materials and methods:**

A total of 40 patients with LE were included in the present study. The patients were randomly assigned to 3 treatment groups: US (n = 13), ESWT (n = 14), and KT (n = 13) groups.

**Results:**

The visual analog scale (VAS) scores significantly decreased in all groups (P < 0.05). Grip strength significantly increased after 8 weeks in only the KT group (P < 0.05). The Patient-Rated Tennis Elbow Evaluation Scale (PRTEE) scores significantly decreased after 2 weeks and after 8 weeks in the US group and ESWT groups, and after 8 weeks in the KT group (P < 0.05). Common extensor tendon (CET) thicknesses significantly decreased after 8 weeks in only the ESWT group (P < 0.05).

**Conclusion:**

The US therapy, KT, and ESWT are effective in reducing pain and improving functionality. However, none of these treatment methods were found to be superior to others in reducing the pain and improving functionality.

## 1. Introduction

Lateral epicondylitis (LE), also known as a tennis elbow, is one of the most common overuse injuries, characterized by pain and tenderness around the lateral epicondyle of the humerus [1,2]. LE is a lesion in the common extensor tendon (CET) that attaches to the lateral epicondyle of the elbow and originates from the fibers of extensor carpi radialis brevis, extensor digitorum, extensor digiti minimi, and extensor carpi ulnaris muscles. The incidence of LE is 1–3% in the general population and is higher in women than in men [2]. Patients suffering from LE usually complain of pain spreading from the lateral side of the elbow to the forearm and this pain affects most activities of daily living (ADL) [1]. LE leads to considerable functional disability as well as loss of performance in occupational and sport activities [2].

Conservative management is recommended as the initial treatment for LE and is considered to be successful in the majority of the patients [3]. A wide variety of conservative applications with different mechanisms of action have been investigated for years in LE [4–8]. One of these conservative applications is ultrasound (US) therapy commonly used in the treatment of the tendon injuries. US is an electrophysical agent which produces deep heat in tissues. Ultrasonic sound waves, which penetrate through the tissue, enhance local blood flow, stimulates inflammatory mediators, and reduce muscle spasm and pain [9].

Extracorporeal shock wave therapy (ESWT) is another noninvasive modality widely used in musculoskeletal pathologies [8,10–12]. ESWT involves the transmission of high-intensity acoustic pressure waves generated by electrohydraulic, electromagnetic, or piezoelectric devices through gel to the target area of ​​the body within a short amount of time. It has been reported that ESWT increases collagen synthesis in tendons, bones, and other soft tissues, accelerates vascularization, and reduces pain [11].

Taping has been used to restrict or facilitate movement in the rehabilitation of the elbows with LE by therapists [13–15]. Unlike other tapes, Kinesio tape, invented by Kenzo Kase, is flexible, sticky, resistant to water, and can remain for 3 to 5 days on the skin. It has been suggested that Kinesio taping (KT) supports weak muscles, corrects joint arrangement, increases blood and lymph circulation, provides proprioceptive input, and reduces pain and muscle spasm [16–20]. In a systematic review published of LE, it was reported that the effects of KT on pain and function in the short term were unclear [21].

The main goals of all conservative applications are to reduce pain and increase functionality but there is a lack of consensus on the most effective conservative application in patients with LE [3]. Sonography is a diagnostic imaging method supporting clinical examination findings to determine the efficiency of treatment and to improve the accuracy of LE diagnosis [22–24]. According to our knowledge, there are few studies comparing the clinical and sonographic findings of the conservative treatments in LE [5–6]. Therefore, the present study aimed to compare the clinical and sonographic effects of KT, ESWT, and US therapy, all of which have been popular in clinical settings.

## 2. Materials and methods

### 2.1. Participants

A total of 40 patients (16 males, 24 females; mean age 48.38 ± 10.35 years; range 24–78 years) who were clinically diagnosed with LE between January 2018 and June 2018 were included in the present study. Inclusion criteria were as follows: (1) pain around the lateral epicondyle during the extension of wrist and fingers against resistance; (2) tenderness over the lateral epicondyle; and (3) symptoms lasting for at least 3 months. Patients with systemic inflammatory disease, fibromyalgia syndrome, cervical radiculopathy, peripheral neuropathy in the upper extremity, ipsilateral medial epicondylitis with limitation, tenderness, swelling, and temperature increase in any joint of the ipsilateral upper extremity; and patients treated with corticosteroids, PRP, or autologous blood injection and physical therapy agents, had upper-extremity surgical intervention, or were pregnant in the previous 6 months were excluded. All patients were informed about the study. Written informed consent was obtained from all patients. This prospective randomized controlled study was approved by the Ethics Committee of the University (3–42/29.03.2017). The demographic data of the patients were also recorded. The patients were also instructed to refrain from any exercise and not to use any painkillers. All patients completed the study.

### 2.2. Outcome measures

The following outcomes were evaluated at baseline, after 2 weeks (at the end of the treatment), and after 8 weeks (in the 6th week after the completion of the treatment). 

Visual analog scale (VAS) was used to assess the intensity of the pain at rest and during ADL. 

Grip strength was measured using a Jamar dynamometer (Baseline, USA) by the researcher, who was blinded to the treatments. The patients were instructed to sit on an armless chair with their shoulders at 0° abduction and in the neutral position, their elbow at 90° flexion, and their forearm in the neutral position. The patients squeezed the dynamometer maximally for 3 s. Three trials were attempted with 60 s of rest between each, and the average of all 3 grips were recorded [25–26]. 

The Patient-Rated Tennis Elbow Evaluation Scale (PRTEE), specifically developed for patients with LE, was used to determine forearm pain and disability. This scale consists of 2 parts, namely pain (5 items) and functional activities (10 items). Each item has a score from 0 (no pain or difficulty in performing a task) to 10 (the worst pain or inability to perform a task). The total score is the combined score of the 2 parts [27,28]. 

Ultrasonography scanning was performed (18/7 MHz linear transducer, Toshiba Aplio 500 Ultrasound System, JAPAN) by a radiologist, who was blinded to the treatments. The thickness/echogenicity of the CET and bony cortex of the lateral epicondyle were assessed during the sonographic imaging (while patients were seated, elbows flexed to 90°, the wrist pronated, and the arm resting on the table).

The patients were randomly assigned to 3 treatment groups using computer generated random numbers.

- US group (n = 13) received a treatment that included hot pack (20 min), transcutaneous electrical nerve stimulation (TENS) (20 min), and US therapy (frequency of 1 MHz, intensity of 1 W/cm2 for 3 min), 5 days a week for 2 weeks.

- ESWT group (n = 14) received a treatment that included hot pack (20 min) and TENS (20 min) 5 days a week for 2 weeks. ESWT (energy density, 0.22 mJ/mm2, pressure 1.4 bar; frequency, 4.0 Hz, pulses, 1500) was applied in 3 sessions for 2 weeks.

- KT group (n = 13) received a treatment that included hot pack (20 min) and TENS (20 min) 5 days a week for 2 weeks. KT was applied once every 2 days for 2 weeks using muscle and fascia correction techniques [18] by an experienced physiotherapist (Figure). The patients were asked to remove the tape before the physiotherapy sessions and taped again afterwards.

**Figure:**
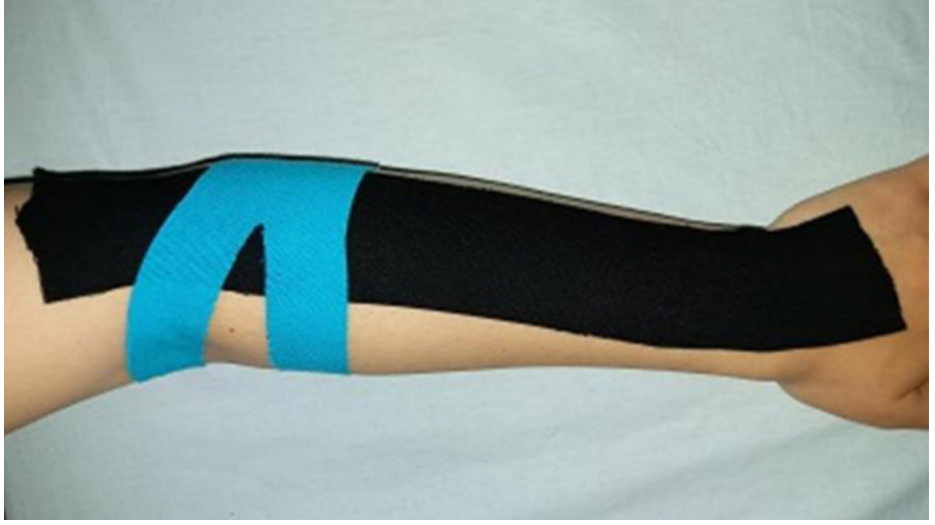
Kinesio taping of lateral epicondylitis.

### 2.3. Statistical analysis

Data were analyzed using SPSS (Version 16.0, SPSS Inc, Chicago, IL). The normality of the dependent variables was checked using the Shapiro–Wilk test. The demographic characteristics of the groups were compared with one-way ANOVA (Table 1). For variables that were normally distributed, a 3 × 4 repeated-measures analysis of variance (ANOVA) was assessed at each evaluation time (baseline, after 2 weeks, and after 8 weeks) for the 3 groups. The Bonferroni test for multiple comparisons was applied to determine the differences between the evaluation times (Table 2). For variables that were not normally distributed, the Kruskal–Wallis test was used to determine differences among the groups (Table 3). The Friedman and Wilcoxon tests were used to compare differences between the evaluation times. All results are shown as mean ± SD. Significance was set at P < 0.05. The sample size was calculated based on a statistical power (1-beta) of 80% and an alpha of 0.05. Eleven participants were required in each group to detect a significant difference in VAS score.

**Table 1:** Table 1. Demographic characteristics of the groups.

Variable	US (n = 13)	KT(n = 13)	ESWT(n = 14)	P
Age (year)	49.62 ± 10.20	47.15 ± 9.87	48.36 ± 11.51	0.839
Sex Male Female	7 (53.8%) 6 (46.2%)	5 (38.5%) 8 (61.5%)	4 (28.6%) 10 (71.4%)	0.424
Body mass index (BMI) (kg/m^2^)	26.39 ± 3.80	27.80 ± 3.72	29.36 ± 6.43	0.298
Dominant hand Righ tLeft	11 (84.6%) 2 (15.4%)	13 (100%)	11 (78.6%) 3 (21.4%)	0.239
Side of involvement Right Left	7 (53.8%) 6 (46.2%)	8 (61.5%) 5 (38.5%)	9 (64.3%) 5 (35.7%)	0.860
Disease duration (month)	2.92 ± 2.98	7.00 ± 6.48	8.07 ± 8.76	0.119

US: Ultrasound; KT: Kinesio taping; ESWT: extracorporeal shockwave therapy.

**Table 2:** Comparison between baseline and posttreatment values of the groups.

Outcome	Group	Baseline X ± SD	After 2 weeks X ± SD	After 8 weeks X ± SD
VAS at rest	US	3.00 ± 2.85	1.54 ± 026	1.31 ± 0.21
	KT	3.23 ± 2.94	2.31 ± 0.30	1.46 ± 0.23
	ESWT	3.21 ± 3.04	0.79 ± 0.14*	1.29 ± 0.25
VAS at ADL	US	7.23 ± 2.61	4.62 ± 2.98*	3.08 ± 3.04*†
	KT	7.46 ± 2.43	4.77 ± 3.08*	3.31 ± 2.89*†
	ESWT	7.79 ± 1.96	4.36 ± 1.64*	3.00 ± 0.30*
Grip strength (kg)	US	31.23 ± 10.94	31.23 ± 9.43	32.00 ± 10.26
	KT	25.85 ± 10.66	28.31 ± 10.88	29.08 ± 10.12*
	ESWT	36.64 ± 23.89	39.57 ± 20.77	40.07 ± 24.32
PRTEE	US	84.38 ± 19.59	62.00 ± 26.95*	59.85 ± 29.91*
	KT	80.92 ± 26.93	67.92 ± 35.10	61.54 ± 32.95*
	ESWT	81.64 ± 32.12	56.29 ± 22.43*	48.14 ± 29.36*
CET thickness (mm)	US	5.16 ± 1.00	5.10 ± 0.90	4.99 ± 0.88
	KT	4.73 ± 0.39	4.60 ± 0.42	4.48 ± 0.46
	ESWT	5.36 ± 0.64	4.82 ± 0.92	4.60 ± 0.74*

X ± SD: mean ± standard deviation; US: ultrasound; KT: Kinesio taping; ESWT: extracorporeal shockwave therapy; VAS: visual analog scale; ADL: activities of daily living; PRTEE: Patient-rated Tennis Elbow Evaluation Scale; CET: common extensor tendon; * significantly different from baseline (P < 0.05); † significantly different from after 2 weeks of treatment (P < 0.05).

**Table 3:** Comparison of the changes of the groups.

Outcome	Time	US(n = 13)X ± SD	KT(n = 13)X ± SD	ESWT(n = 14)X ± SD	P
VAS at rest	Baseline, after 2 weeks	1.46 ± 0.32	0.92 ± 0.33	2.42 ± 0.29	0.546
	Baseline, after 8 weeks	1.69 ± 0.32	1.76 ± 0.29	1.92 ± 3.51	0.873
	After 2 weeks, after 8 weeks	0.23 ± 0.10	0.84 ± 0.10	050 ± 0.19	0.112
VAS at ADL	Baseline, after 2 weeks	2.61 ± 0.33	2.69 ± 2.83	3.42 ± 1.60	0.397
	Baseline, after 8 weeks	4.15±0.40	4.15±2.85	4.78 ± 3.06	0.801
	After 2 weeks, after 8 weeks	1.53 ± 0.19	1.46±0.96	1.35 ± 0.24	0.985
Grip strength (kg)	Baseline, after 2 weeks	0.00 ± 0.40	2.46 ± 0.40	2.92 ± 0.71	0.272
	Baseline, after 8 weeks	0.76 ± 0.42	3.23 ± 0.45	3.42 ± 0.60	0.346
	After 2 weeks, after 8 weeks	0.76 ± 0.22	0.76 ± 0.17	0.50 ± 0.07	0.956
PRTEE	Baseline, after 2 weeks	22.38 ± 2.22	13.00 ± 2.19	25.35 ± 24.11	0.679
	Baseline, after 8 weeks	24.53 ± 2.63	19.38 ± 2.16	33.50 ± 3.56	0.385
	After 2 weeks, after 8 weeks	2.15 ± 1.06	6.38 ± 1.29	8.14 ± 2.52	0.450
CET thickness (mm)	Baseline, after 2 weeks	0.06 ± 0.08	0.12 ± 0.02	0.53 ± 0.10	0.182
	Baseline, after 8 weeks	0.16 ± 0.07	0.24±0.03	0.76 ± 0.73	0.075
	After 2 weeks, after 8 weeks	0.10 ± 0.02	0.12 ± 0.02	0.22 ± 0.06	0.739

X ± SD: mean ± standard deviation; US: ultrasound; KT: Kinesio taping; ESWT: extracorporeal shockwave therapy; VAS: visual analog scale; ADL: activities of daily living; PRTEE: Patient-rated Tennis Elbow Evaluation Scale; CET: common extensor tendon.

## 3. Results

The demographic characteristics of the groups are presented in Table 1. A total of 40 participants were included in the study; 13 were randomized to the US group, 13 to the KT group, and 14 to the ESWT group. The age, sex, body mass index, dominant hand, side of involvement, and disease duration did not differ between the groups (P > 0.05). The VAS scores at rest significantly decreased from baseline (3.21 ± 3.04) to after 2 weeks of treatment (0.79 ± 0.14) in only the ESWT group (P = 0.012). However, there were no significant differences in the US (P > 0.05) and KT (P > 0.05) groups (Table 2). The VAS scores during ADL significantly decreased after 2 weeks in the US, KT, and ESWT (P = 0.018, 0.010, 0.001, respectively) and after 8 weeks in the US, KT, and ESWT groups (P = 0.008, 0.003, 0.002, respectively) (Table 2).

Grip strength significantly increased after 8 weeks compared to baseline in only the KT group (P = 0.021) (Table 2).

The PRTEE scores significantly decreased after 2 weeks in the US and ESWT groups (P = 0.010, 0.005, respectively) and after 8 weeks in the US, KT, and ESWT groups (P = 0.017, 0.022, 0.011, respectively) (Table 2).

The CET thicknesses significantly decreased after 8 weeks in only the ESWT group (P = 0.006) (Table 2).

No significant differences between the groups were observed for the VAS scores at rest and during ADL, grip strength, PTREE, and extensor tendon thicknesses compared to baseline among the 3 groups (P > 0.05) (Table 3).

## 4. Discussion

The results of the present study showed that all treatment interventions significantly reduced pain intensity during ADL at the end of the treatment and at 6 weeks following the completion of the treatment. The functional status of the arms of the patients determined by PRTEE significantly improved at the end of the treatment in the ESWT and US groups and after 6 weeks following the completion of the treatment in the KT group. Another finding of this study is the improvement in grip strength of only the KT group after 6 weeks following the completion of the treatment. However, none of the treatment methods were found to be superior to others.

US therapy has been used at different modes, intensities, and durations according to tissue depth and the type of the injury in soft tissue lesions such as tendinitis, tenosynovitis, epicondylitis, and bursitis [29]. In the present study, US therapy showed similar improvements in terms of the pain and functional status of the patients with LE consistent with previous studies in the literature. Öken et al. [30] applied US therapy at a frequency of 1 MHz and intensity of 1.5 W/cm2 for 5 min for 10 sessions in a study in which the effects of laser therapy, bracing, and US therapy were compared. They demonstrated that all 3 treatment methods decreased pain at the second week of the treatment according to baseline. In addition, the effects of US therapy and laser therapy continued for 6 weeks after treatment. Although all patients were given strengthening and stretching exercises, they found no significant difference in patient grip strength in the US group. Lizis [31] investigated the effects of 5 sessions of ESWT (pressure, 2.5 bar; frequency, 8 Hz; energy density, 0.4 mJ/mm2) and 10 sessions of US therapy (intensity, 0.8 W/cm2 and frequency, 1 MHz) applied 3 times per week. It was found that ESWT alleviated pain more than US therapy immediately and for 3 months after the treatment. In the present study, the patients were given a lower pressure, density, and frequency of ESWT for 3 sessions. Therefore, the difference between ESWT and US therapy could not be detected. It is suggested that ESWT stimulates recovery via the trigger of inflammation due to mechanical irritation caused by shock waves [32] and increases blood circulation in the focused tissue [33]. In the treatment of LE, conflicting results have been reported because of the number of sessions and the dose of the treatment applied [34]. In contrast to the present study, there are 2 studies that included ESWT and US therapy as part of treatment and found a significant improvement in patient grip strength with LE [6,35]. Yalvaç et al. [35] applied 10 sessions of US therapy and 3 sessions of ESWT to the subjects. Their results showed that pain intensity decreased and grip strength and PRTEE scores improved after treatment and in the 1-month follow-up in both groups. Gündüz et al. [6] revealed that VAS scores were significantly reduced within the first month, and 3 and 6 months after 10 sessions of ESWT, local steroid injection, and classic physiotherapy in LE. The classic physiotherapy group received not only US therapy but also hot pack and friction massage. However, the grip strength improved after 1 month in 3 groups and continued in the sixth month of the treatment in ESWT group. In the present study, the absence of a significant increase in patient grip strength may be attributed to the inadequate dose of US therapy and ESWT. 

In this study, the grip strength improved only in the KT group after 6 weeks following the completion of the treatment. The Kinesio tape expands the distance between the muscle and the interstitial area and lifts the skin upwards by creating microcurves on the skin. Thus, lymphatic circulation is accelerated, and the stimulation of the subcutaneous pain receptors is prevented [36]. Thanks to these properties, KT may increase the range of motion without pain and allow the muscle to produce more force. Furthermore, KT stimulates cutaneous mechanoreceptors, creates proprioceptive feedback, and increases the activation of motor units [17]. There have been variable results about the effect of KT on grip strength in patients with LE. Cho et al. [16] revealed that KT reduced pain, increased grip strength, and improved functionality immediately after taping. Dilek et al. [13] demonstrated that VAS at rest, grip strength, and PRTEE scores improved within 2 and 6 weeks following a 2-week application of KT. However, their study did not include any placebo or control group. Eraslan et al. [14] investigated the effects of KT, ESWT, and classic physiotherapy for 3 weeks. The patients received a cold pack and TENS treatment for 15 sessions and a home exercise program including stretching and strengthening exercises in addition to KT and ESWT. The pain, grip strength, and functionality improved according to baseline in 3 groups. However, the KT group was found to be superior to the classic physiotherapy and ESWT. In the present study, the patients did not receive any exercise intervention. Exercising may contribute to the increase in grip strength. Giray et al. [37] compared KT combined with exercises, sham taping, and exercises only. Taping with exercises was more effective than the others in the reduction of pain and disabilities of the upper limb after 4 weeks following treatment. In contrast to the results of the present study, they did not find any improvement in the grip strength. Au et al. [38] reported nonsignificant results in pain intensity, grip strength, and electromyographic activity immediately after taping. Their study examined the effects of placebo, facilitatory KT, inhibitory KT, and sham KT. They suggested that muscle fatigue may have affected grip strength due to the short washout period in the crossover study. Another limitation of their study was a shorter application period compared to the present study. Shakeri et al. [39] showed that VAS scores during activity and functional disability decreased immediately or after a 3-session KT application and grip strength did not change significantly. The authors asserted that testing in extension of the elbow reduced muscle tonus. In the present study, grip strength was measured with the elbow flexed at 90°. It has been suggested that grip strength measured at 90° of elbow flexion is stronger than elbow extension in patients with LE [40]. Their study compared KT with tension and placebo (KT without tension). As a result, KT studies can produce variable results in improving grip strength depending on factors such as taping technique, application period, and measurement method of grip strength in LE. 

It is known that ultrasonographic measurement is an important diagnostic method that supports the clinical examination findings in the diagnosis of LE [22–24]. In the present study, although CET thickness improved in all groups, a significant decrease was found after 6 weeks following the completion of the treatment in only the ESWT group. The results of this study are important because, to our knowledge, there is only one study that evaluated the effectiveness of ESWT by ultrasonographic method in LE. Gündüz et al. [6] applied ESWT in patients with LE. In their study, CET thicknesses did not change significantly compared with the baseline measurements at the 6-months follow-up in the ESWT, local steroid injection, and classic physiotherapy groups. They suggested that further measurement following the 6-month follow-up was needed to achieve a significant result in CET thickness. 

A limitation of the present study was that the length of the follow-up was short compared to previous studies in the literature [6,23,24]. Another limitation of the present study was that there was no exercise intervention in addition to the treatment methods applied. Grip strength may be increased by strengthening the forearm muscles.

The results of the present study showed that US therapy, KT, and ESWT were all effective in reducing pain and improving functionality in patients with LE and none of these treatment methods were superior to each other. However, KT was found to be the most effective treatment method in the improvement of the grip strength. ESWT can lead to an improvement in extensor tendon thicknesses in the long term. 

## Informed consent

All participants signed an informed consent form before participating in the study. This study was approved by the Ethics Committee of the Karabük University (3–42/29.03.2017).
